# Synthetic RNA biology

**DOI:** 10.1080/15476286.2024.2335746

**Published:** 2024-04-14

**Authors:** Beatrix Suess

**Affiliations:** Department of Biology, Synthetic RNA Biology, TU Darmstadt, Darmstadt, Germany; Centre for Synthetic Biology, TU Darmstadt, Darmstadt, Germany

Synthetic biology has recently grown out of its infancy as primarily DNA- and protein-centred research. However, proteins remain the dominant biobrick. It should then come as no surprise that more and more exciting and innovative research using RNA is being reported. These highlights reveal the enormous potential of synthetic RNA biology, promising a variety of exciting applications and, most importantly, leading to a better understanding of diverse biological processes.

In synthetic biology, organisms and biological systems with novel properties and functionalities are created by assembling biological components, some of which do not even exist in nature. RNA engineers not only take advantage of the fact that RNA can interact in a very specific and predictable way through complementary base pairing, but also exploit the ability of RNA to form highly complex structures and thus bind a wide variety of target molecules. Many of these RNA elements exist in nature, but can also be selected *de novo*. It is therefore not surprising that researchers are now recognizing the enormous potential of RNA for the exciting and rapidly developing field of synthetic biology. This special issue is an attempt to summarize recent developments in synthetic RNA biology, highlighting engineering and design strategies at various scales from the molecular to the circuit to the cellular level, and summarizing the enormous spectrum of potential applications in biotechnology and therapeutic approaches.

## RNA-mediated conditional control of gene expression is not limited to the control of promoter activity

Synthetic RNA-based regulatory systems that respond to internal or external signals to control protein-encoded output are gaining increasing attention with the recent rise of mRNA therapeutics, the capacity of these systems as building blocks to construct synthetic regulatory networks, or as sensing devices. RNA-based approaches are versatile as they are not limited to the control of promoter activity. In this special focus, different strategies for RNA-based regulatory systems will be discussed. They use RNA binding proteins (RBPs) [1] and small molecules [2,3] as triggers, or RNA molecules expressed in the cell that affect a target RNA by base pairing [4–6]. Thereby multiple mechanisms can be used to regulate gene expression, ranging from the control of translation and splicing to the precise control of mRNA stability [1–6].

RNA-RBP pairs can be used to create regulatory systems and are derived either from natural counterparts or from RNA partners newly generated by an *in vitro* selection process called SELEX. However, the number of available RNA partners is still limited, which currently inhibits multiplexing [1]. One way of solving this problem is by redesigning existing RNA-RBPs. One of the most important applications of these systems is their potential to track the concentration of specific intracellular proteins. Proteins are responsible for a wide range of biological phenomena within cells. Variations in protein expression underlie cellular states and identities. Therefore, sensors that detect proteins and generate measurable outputs are indispensable tools for cellular engineering applications [1].

An alternative approach to the regulation of gene expression is the use of synthetic riboswitches. In this case, the trigger is not a protein but a small molecule. While natural riboswitches are almost exclusively found in bacteria, synthetic riboswitches have been developed for various other organisms, including mammalian cell lines. Using the tetracycline-binding aptamer as an example, different strategies for the construction of synthetic riboswitches have been summarized, but also the limitations that not all *in vitro* selected aptamers can be used have been addressed. Excellent binding properties as well as structural flexibility are required [2]. For the design of such switches, self-cleaving ribozymes are often used. However, when applied in mammalian gene expression systems in particular, the results can often be unexpected. A variety of factors, such as the surrounding sequence context, the positioning of the ribozyme, or the ribozyme variant used, influence the activity and thus its performance, which was addressed in a comparative study [3].

Beside proteins and small molecules, RNA itself can also be the trigger [4]. This enables the design of RNA-sensing systems that can help to identify specific cell types, but also manipulate cell fate in a cell type-specific manner. Synthetic devices can be engineered that sense the cellular level of a transcript, such as miRNAs, and reprogram the output depending on the miRNA signature of the cell [7]. A milestone study in 2014 reported about so-called toehold switches that control translation initiation in *Escherichia coli* based on RNA strand displacement [8]. This finding opened up the opportunity to sense RNAs that do not already have a regulatory function. These switches consist of a switch RNA that encodes for an output and a trigger RNA that can activate the expression of the switch RNA. In the switch RNA, a stem loop blocks a functional site, e.g. a ribosomal binding site or intrinsic terminators of transcription. The trigger then binds a single-stranded region to open up the sequestration hairpin, exposing the functional site. The system works in bacteria, but also in cell-free systems and has also been adapted for eukaryotic systems by controlling internal ribosomal entry sites or RNA editing [4].

It is becoming increasingly clear that the dynamics of the molecular processes described here are becoming more and more important. The folding of RNA molecules plays a central role not only in the control of various cellular processes, but also in the targeted design of RNA-based synthetic regulatory systems. The focus on RNA folding allows the development of new concepts for programming and reprogramming of RNA and the optimization or development of new functions in biotechnology. This is not only limited to the molecular level by engineering individual parts and devices, but also finds application at the circuit level, where advances in controlling the interaction of multiple RNAs are leading to the engineering of new dynamic systems, including RNA logic circuits and feedback loops. It extends to the cellular level, where higher order RNA assemblies are being engineered into new classes of biomaterials, biomaterial condensates, or core components of extracellular vesicle signalling and synthetic cells [8].

## To see and to be seen – RNA as a sensing and visualization device

RNA molecules have excellent sensing capabilities, enabling them to detect and respond to intracellular protein or RNA levels, but also to recognize small molecules like environmental pollutants or altered metabolites. However, given the enormous potential of RNA as a molecular sensor, it is crucial that RNA molecules can be easily traced. Here, too, enormous efforts have been made. With the discovery of GFP, molecular biology was revolutionized. Gene expression studies became much easier and more quantifiable, and protein localization could be visualized in cells and tissues. The counterpart for RNA was born with the discovery of spinach, an aptamer that binds a fluorophore, which led to the new and rapidly growing field of developing RNA-based imaging probes. These days, there is a whole ‘vegetable garden’ of various aptamers that glow in different colours, including spinach, broccoli, corn, squash, chilli, mango and others [9]. A major drawback is that many of these aptamers do not retain their fantastic properties when they are expressed under cellular conditions. Applications are therefore often limited. Continuous efforts are being made to improve the folding of these aptamers so that they can also be used in cells, especially in Mammalia. These include classical approaches such as rational mutagenesis, but also the use of stabilizing ligands, scaffolds partially derived from natural RNA structures, or the use of circular RNAs [9]. These novel approaches, which are not limited to imaging RNA aptamers, make synthetic RNA-based concepts approaches, including therapeutic applications, much more feasible.

## RNA synthetic biology towards therapeutic application

With the introduction of RNA vaccines during the COVID-19 pandemic, RNA became recognized by the public as a molecule with therapeutic potential. This potential extends far beyond its use as a vaccine. Recent advances in the field of RNA origami and RNA nanoscaffolding have led to the creation of complex arrangements that can even be expressed in living cells. Starting from the assembly of simple RNA motifs into higher order structures, RNA origami of several thousand bases can now be predicted and designed to form RNA nanopores, tiles or filaments. These nanostructures can be functionalized with aptamers as anchoring sequences, binding partners or catalytic moieties [10]. They then can be used for therapeutic and diagnostic purposes, as scaffolds or for functional delivery. As an example, RNA nanostructures harbouring siRNAs, RNA aptamers, synthetic miRNAs, nanoscaffolds and conjugates for drug delivery are described in the context of cancer treatment [11]. Nevertheless, there are several challenges to using these RNAs *in vivo*. Some, such as the stability of RNA *in vivo*, can already be overcome by chemically modified nucleotides, while others, like the circulation time in blood or the biodistribution in the body, require further research. Overall, RNA nanostructures have great potential for medical applications, and more research on this topic will help to unlock their full capabilities.

Finally, I would like to express my wish that the reader of this special issue will enjoy the collection of articles compiled. I hope that the reader will experience the same fascination that I did while reading these articles and agree with me that there is enormous potential in this new discipline that has only just begun to be tapped.
